# Evaluation of bowel cancer registration data in England, 1996–2004

**DOI:** 10.1038/sj.bjc.6605321

**Published:** 2009-09-22

**Authors:** A M Jones, E Morris, J Thomas, D Forman, J Melia, S M Moss

**Affiliations:** 1 Cancer Screening Evaluation Unit, Sir Richard Doll Building, Institute of Cancer Research, 15 Cotswold Road, Sutton, Surrey, SM4 5NG, UK; 2 Cancer Epidemiology Group, Northern and Yorkshire Cancer Registry and Information Service, St James's Institute of Oncology, University of Leeds, Level 6, Bexley Wing, Alma Street, Leeds, LS9 7TF, UK

**Keywords:** colorectal cancer, registries, screening, stage, incidence, trends

## Abstract

**Background:**

The National Health Service (NHS) bowel cancer screening programme (BCSP) was initiated across England in April 2006. To determine the feasibility of using national cancer registration data to assess the impact of the BCSP on stage-specific incidence, we studied trends in the incidence rates of colon (ICD10 C18) and rectosigmoid junction and rectum (ICD10 C19–C20) cancers and the completeness of data on Dukes stage in England.

**Methods:**

Data were obtained from all nine cancer registries for the period 1996–2004, before the introduction of the BCSP, in men and women aged 50–79 years.

**Results:**

Overall, incidence rates declined by 1% per year in the 9 years before the introduction of the BCSP (*P*<0.001). Dukes stage was recorded for 60% of all registrations but this varied between regions and over time. Only four registries had completeness of 74% or more. Registrations with unknown Dukes stage decreased from 1996 to 2000, and then increased during 2001–2004 affecting trends in stage-specific incidence.

**Conclusion:**

To study the impact of the BCSP on stage-specific incidence, regional variations in data completeness need to be addressed.

Screening by fecal occult blood testing (FOBt) has been shown to significantly reduce mortality from bowel cancer in randomised controlled trials. A trial in Nottingham, UK, showed a 15% reduction in bowel cancer mortality in those randomised to the intervention arm and offered biennial FOBt screening at a median of 7.8 years follow-up (Hardcastle *et al*, 1996). In 2000, a pilot study (UK Colorectal Cancer Screening Pilot Group, 2004; Weller *et al*, 2007) was established to assess the feasibility of population-based screening for bowel cancer in the United Kingdom using FOBt. The results of the pilot supported roll out of the National Health Service (NHS) bowel cancer screening programme (BCSP), which was initiated across England in April 2006 (NHS Bowel Cancer Screening Programme, 2008). It is anticipated that full coverage will be achieved by the end of 2009. A total of five million men and women aged 60–69 years will be offered screening by FOBt every 2 years. An extension of the age range up to 75 years is planned from April 2010.

The main aim of the screening programme is to reduce bowel cancer mortality by detecting bowel cancers in asymptomatic people at an early stage when treatment is most effective. Screening also detects adenomas, which could potentially develop into cancers, and removal of these will reduce the risk of developing bowel cancer in affected individuals. The expected reduction in bowel cancer mortality is unlikely to be observable for many years, and it is therefore important to study interim measures of performance to assess whether the screening programme is on track to achieve the expected benefit. Monitoring interim performance measures will also ensure that the information being collected is appropriate, adequate and of high quality.

Survival of bowel cancer patients decreases markedly with increasingly advanced stage of disease. Dukes classification (Dukes, 1949) is widely used to describe the staging of bowel cancer; a change in the distribution of Dukes stage is therefore a key performance indicator. A reduction in the incidence rate of late stage (Dukes C & D) cancers would be expected to precede a reduction in bowel cancer mortality because of screening. Studies of the local impact of the bowel cancer Pilot in Coventry and north Warwickshire (Goodyear *et al*, 2008) found a significantly lower proportion of advanced cancers in those detected by screening compared with those not screen detected.

A previous assessment of bowel cancer registration data for the Oxford region (Green *et al*, 2007) concluded that incomplete registry data and changing recording practices may affect future evaluation of bowel cancer screening.

The aim of this paper is to report the trends nationwide in cancers of the colon (ICD10 C18) and of the rectosigmoid junction and rectum (ICD10 C19 and C20) for England for the period 1996–2004 before the introduction of the national screening programme, and to assess the completeness and quality of data from all registries.

## Materials and methods

Individual data on colorectal cancer registrations for men and women aged 50–79 years over the period 1996–2004, were collected from the nine English cancer registries existing during this period: Eastern Cancer Registration and Information Centre (ECRIC), Merseyside and Cheshire Cancer Registry (MCCR), North Western Cancer Registry (NWCR), Northern and Yorkshire Cancer Registry and Information Service (NYCRIS), Oxford Cancer Intelligence Unit (OCIU), South West Cancer Intelligence Service (SWCIS), Thames Cancer Registry (Thames), Trent Cancer Registry (Trent) and West Midlands Cancer Intelligence Unit (WMCIU). The National Minimum Dataset(United Kingdom Association of Cancer Registries, 2008) for colorectal cancer registrations includes: patient unit number, NHS number, name, postcode, sex, date of birth, date of diagnosis, site of primary neoplasm, morphology, Dukes stage and grade of tumour. These data were collated by NYCRIS and anonymised before being sent to the Cancer Screening Evaluation Unit.

A number of additional variables derived by NYCRIS were included in the extract supplied. These were tumour identification code, number of tumours, postal district, and a variable indicating whether the tumour was registered in two registries. In addition a composite Dukes stage was generated. Cancer registries record up to three types of stage for colorectal tumours: Dukes, pathological TNM and clinical TNM. These three different staging systems have been combined so that just Dukes stage is reported. To do this the following rules were applied. Firstly, all pathological and clinical TNM stages were converted to a Dukes stage and pathological TNM or Dukes were taken as the ‘gold standard’ staging information. However, if both a pathological TNM and a Dukes stage had been reported, but they did not agree the highest stage available was used. In addition if no pathological TNM or Dukes was available but a clinical TNM stage was available, then clinical stage was used. Finally if no Dukes, pathological TNM or clinical TNM was available then the tumour was classed as unstaged.

Duplicate registrations can occur when diagnosis takes place in one registry but treatment occurs in another, either because an individual has moved between diagnosis and treatment or because they were treated in a hospital in a different region. Before analysis, any duplicate registrations that could be identified and that were not true multiple primaries were removed.

Crude age- and sex-specific bowel cancer incidence rates per 100 000 persons were calculated in 5-year age groups for the whole of England, by cancer site and by Dukes stage. The national population counts for the denominators were obtained in 5-year age and sex subgroups for the years 1996–2004 from the Office for National Statistics. Crude age- and sex-specific bowel cancer incidence rates per 100 000 were also calculated for populations covered by cancer registries. Cancer registry population counts used in the denominator were provided in 5-year age and sex subgroups for the years 1996–2004 by UKACR (United Kingdom Association of Cancer Registries, 2009). These population counts of 50 to 79 year olds for each year were estimated for the eight cancer registries as they exist in 2009 by their geographical catchment area: ECRIC, the merged MCCR and NWCR called the North West Cancer Intelligence Service (NWCIS), NYCRIS, OCIU, SWCIS, Thames, Trent and WMCIU. The crude sex-specific incidence rates were adjusted for age using the European population as standard (Doll and Cook, 1967). Poisson regression modelling was used to investigate how incidence rates differ by age, sex, year of registration and cancer registry using the STATA (version 9.2) statistical package.

Completeness of Dukes stage was measured as the proportion of all registered cases, which were categorised as known stage (A–D). Tumour grade completeness was measured as the proportion of cases categorised as known grade (well, moderate, poor or undifferentiated). Completeness of TNM stage was not studied separately. It was not part of the National Minimum Dataset (United Kingdom Association of Cancer Registries, 2008) over the period of study and there was insufficient information to permit valid analysis either by registry or nationally.

The 2004 national peer review of cancer registry measures (Department of Health, 2005) recommended that registries should have Dukes stage information for a minimum of 74% of bowel cancer cases. In line with these recommendations, stage-specific incidence rates were first calculated using the full dataset and then restricted to cancer registries, which had a minimum of 74% completeness for the period 1996–2004. Incidence rates by stage are reported here from the restricted dataset only.

## Results

Between 1996 and 2004, a total of 177 379 individual bowel cancers in men and women aged 50–79 years were registered in England. There were 109 196 cancers of the colon and 68 183 cancers of the rectosigmoid junction and rectum. Crude incidence rates for all sites (ICD10 C18–C20) for men and women were 172 per 100 000 and 117 per 100 000 in 1996, and 163 per 100 000 and 105 per 100 000 in 2004, respectively.

Table 1 shows that the mean age at diagnosis was slightly, but significantly, lower in men (68.4 years) than in women (69.0 years) (*P*<0.001) Age-specific incidence rate ratios of men/women shows that the rate was higher in men than women in each age group, for example, in 2004 for age groups 50–54, 55–59, 60–64, 65–69, 70–74 and 75–79 years the ratios were 1.38, 1.46, 1.68, 1.64, 1.71 and 1.69, respectively. Mean age at diagnosis for cancers of the rectosigmoid junction and rectum was significantly younger than for cancers of the colon (68.0 years compared with 69.1, respectively) (*P*<0.001).

**Figure 1:**
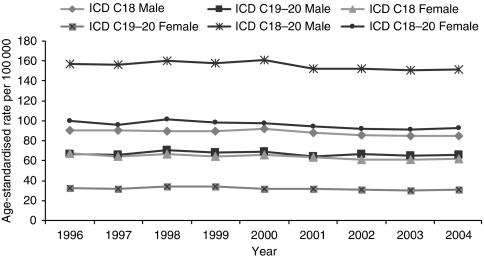
European age-standardised incidence rates of bowel cancer (ICD10 C18–C20), by sex, and by sex and site in 50–79 year olds: England 1996–2004.

Age-standardised incidence rates of bowel cancer declined between 1996 and 2004 (Figure 1). The results of a Poisson regression analysis showed that after adjustment for age and cancer registry, incidence rates declined by an average of 1% (*P*<0.001) each year for both men and women; when sex was included in the model, the risk of bowel cancer was 39% lower for women compared with men (*P*<0.001).

**Figure 2:**
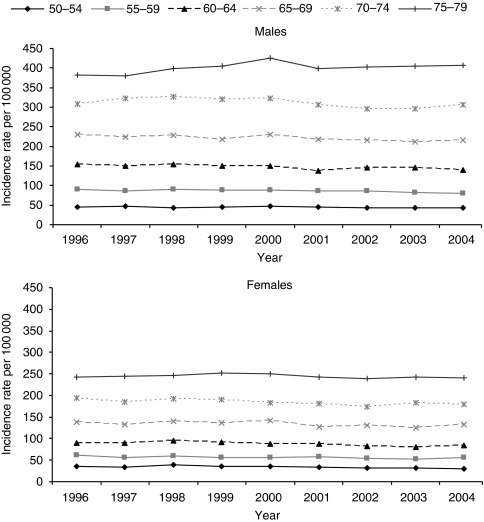
Age-specific incidence rates of bowel cancer (ICD10 C18–C20), by sex: England 1996–2004.

Age and sex-specific incidence rates also declined significantly (*P*<0.001) by 1% each year for all age–sex groups except for the 75–79 year olds in which there was no change in women and a slight increase in men (Figure 2). Both incidence rates and their decrease with time varied between cancer registries.

Age-standardised incidence rates of bowel cancer for the study period as a whole varied considerably between cancer registries for men, but less so for women. Incidence rates in men varied from 111 per 100 000 in the Thames Cancer Registry region to 140 per 100 000 in the NYCRIS region. In women, incidence rates varied from 70 per 100 000 in the Trent Cancer registry region to 83 per 100 000 in OCIU region. Men in the ECRIC region and women in the WMCIU region experienced the largest drops in rates between 1996 and 2004 of 16 and 17 per 100 000, respectively. In contrast, incidence rates for both men and women in the SWCIS and Trent Cancer registry regions increased slightly (between 0.4 and 1.4 per 100 000) but these changes were not significant (*P*>0.05).

### Site-specific incidence

Age-standardised incidence rates of colon cancer (ICD10 C18) were higher than those for cancers of the rectosigmoid junction and rectum (ICD10 C19 and C20) (Figure 1) and declined steadily between 1996 and 2004. Poisson regression analysis showed that after adjusting for age, sex and cancer registry, incidence rates for colon cancer were higher than those for rectosigmoid junction and rectum cancers (*P*<0.001) (35% higher in men and 106% higher in women). There was a 1% yearly decline in incidence rates in colon cancers in both sexes and for rectosigmoid junction and rectum cancers in women (*P*<0.001).

### Dukes stage and grade

Using the composite Dukes stage described in the Materials and Methods section, Dukes stage was recorded for 60% of all registrations (Table 2). Completeness was slightly higher for colon cancers compared with those of the rectosigmoid junction and rectum (62 and 58%, respectively). Completeness of stage did not vary by gender but it was lower in those aged 75–79 years compared with the 50–54 year olds (60 *vs* 63%, respectively for colon cancers and 54 *vs* 61% for the rectosigmoid junction and rectum). Completeness varied between cancer registries; ECRIC, NYCRIS, OCIU and WMCIU had the highest levels (⩾74%, Table 2). Completeness in all but one registry improved over time between 1996 and 2000.

**Figure 3:**
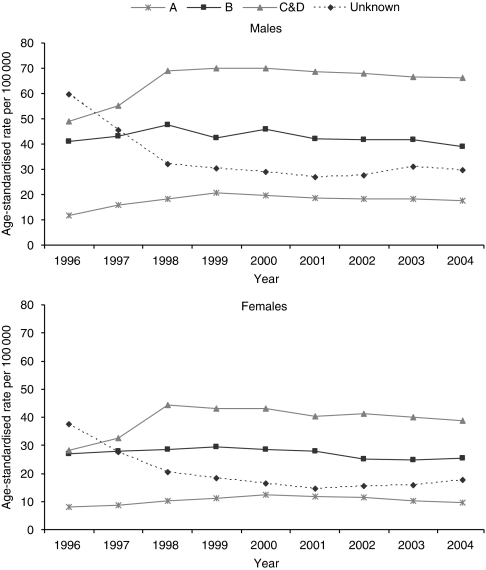
European age-standardised incidence rates of bowel cancer (ICD10 C18–C20) in Eastern, Northern and Yorkshire, Oxford and West Midlands cancer registries by Dukes stage and sex: England 1996–2004.

Figure 3 displays the age-standardised incidence rates, by Dukes stage, after restricting the data to those four cancer registries with stage completeness of 74% or above. Incidence rates for all known stages, but less so for Dukes stage B, increased during the period 1996 to 2001, for both men and women, and were highest for Dukes stage C&D. However, from 2001 the rates declined. Incidence rates of unknown Dukes stage declined significantly (*P*<0.001) over the period 1996–2001 and this is likely to account for most of the increase in rates for known stages during this period. After 2001, the rate of unknown stage cancers increased, coinciding with the decline in the rate of cancers with known stage.

Tumour grade was recorded for 54% of all registrations, but reliable and complete grades were recorded for only 46% of all registrations.

## Discussion

We have analysed bowel cancer incidence rates and completeness of stage data from all cancer registries in England for the period before the introduction of the national screening programme. The underlying trend in bowel cancer incidence rates in England, for both men and women, showed a steady and significant decline over the 9-year period before the introduction of the screening programme, averaging around 1% per year. Colon cancers accounted for 62% of all registrations (57% for men and 67% for women). Between 1996 and 2004, incidence rates declined across all cancer sites and all age groups excluding the oldest age group and rectal cancers in men.

A significant finding was the incompleteness of data on stage, with overall 40% of registrations having no Dukes stage information. Regional variation in completeness partly reflects differing recording practices and the extent to which registries are able to verify metastatic disease and avoid recording the Dukes stage as ‘unknown’. Of particular concern was the change over time in the rate of cancers with stage unknown, as such changes will affect the rate of cancers of known stage. In the period studied, the rate of cancers with stage unknown decreased until 2001 and then showed a slight increase until 2004. As noted elsewhere poor levels of completeness will hamper future analyses of stage registration within the screening programme framework (Green *et al*, 2007). One solution would be to restrict analyses to data from registries, which have at least 74% completeness in line with the 2004 National Cancer Peer Review Standards (Department of Health, 2005). The recent increase in cancers with stage unknown will be partly due to increasing use of neo-adjuvant treatments in the management of rectal tumours, and the lead registry for colorectal cancer NYCRIS (in collaboration with the UKACR) has recommended that registries should switch from collecting Dukes stage to TNM (Version 5) and that registry data systems should incorporate a *y* prefix to TNM stages to indicate those patients who underwent neo-adjuvant treatment. This level of detail would enhance the data quality and future analysis.

A strength of this paper is that it presents results for all registries in England. A limitation of our analysis is that cancer registry population counts were only available based on 2009 registry boundaries. In the years following the end of our study period there were a number of changes to registry catchment areas, but we were only able to calculate registry incidence rates for registries based on the 2009 boundaries. However, aggregated cancer registry population counts, by year, closely match the national counts.

This paper has not investigated ascertainment levels of bowel cancer registrations between registries. However, the trends in incidence rates indicate that it is unlikely that there were variations in case ascertainment over time.

Monitoring the performance and success of the screening programme will be an ongoing exercise. There are five screening hubs in England, each responsible for co-ordinating the screening programme in around 20 local screening centres (NHS Bowel Cancer Screening Programme, 2008). The BCSP has been rolled out gradually by PCT and it may be possible to study future incidence according to the timing of the introduction of screening. Full coverage is due to be achieved by December 2009. Over the study period there were 257 395 bowel cancers in all ages in our dataset, of these 23% were in the screening age range (60–69 years).

The full effect of the programme on mortality from bowel cancer is not likely to emerge until around 2020. However, it has recently been demonstrated that the benefit of flexible sigmoidoscopy screening on bowel cancer mortality could be estimated by predicting colorectal cancer mortality based on stage-specific incidence thus reducing the required follow-up time by around 3 years (Cuzick *et al*, 2007). The approach requires reliable stage data and it was emphasised that the routine collection of such data should be a priority for cancer registries.

The screening programme should also lead to the detection and removal of adenomas before they develop into cancers, which in turn could lead to an overall reduction in the underlying bowel cancer incidence rate. However, there is no indication so far from the Nottingham RCT, whose protocol the screening programme follows, that such a reduction will be observed (Scholefield *et al*, 2002) and any reduction is likely to take around 15–20 years.

National FOBt screening programmes have been implemented in several countries, either in full or part (Benson *et al*, 2008), but most are recent developments, and few results about the effect on incidence have been published. In Japan, where the screening programme commenced in 1993, an increase in age-standardised incidence rates of screen detected bowel cancer was observed in the first 4 years; compared with 1988–1992, incidence increased 1.4 and 1.2 times for men and women, respectively (Minami *et al*, 2006).

Changes in age-specific bowel cancer incidence will provide an early indication of the effect of the programme, although they will not provide evidence of an eventual impact on mortality. In the first few years after the introduction of the programme, the incidence rate would be expected to increase because of the detection of prevalent cases by screening. The incidence rate would then be expected to return almost to the background level, except in the youngest age group offered screening in which prevalent screens will still be taking place (Quinn *et al*, 1995). There should also be a slight fall in incidence in the age group just above the screening age range due to earlier diagnosis of cancers that would otherwise have occurred at these ages.

In conclusion, analyses of stage-specific incidence rates before the introduction of the BSCP will need to take into account Dukes stage completeness. The effect of earlier diagnosis because of programme awareness and changes in registration practices should also be considered.

## Figures and Tables

**Table 1:** Malignant neoplasms of the colon (ICD10 C18) and of the rectosigmoid junction and rectum (ICD10 C19–C20): England 1996–2004

	**Cancer site**					
	**ICD10 C18**			**ICD10 C19–C20**		
	**Total**	**Male patients**	**Female patients**	**Total**	**Male patients**	**Female patients**
Number of registrations	109 196	59197	49999	68 183	43905	24278
Mean age at diagnosis (years)	69.1	68.9	69.3	68.0	67.8	68.4
						
*Dukes stage*						
A	6509 (6%)	3605 (6%)	2904 (6%)	8107 (12%)	5069 (12%)	3038 (13%)
B	26 873 (25%)	14 264 (24%)	12 609 (25%)	12 401 (18%)	8176 (19%)	4225 (17%)
C	25 283 (23%)	13 597 (23%)	11 686 (23%)	14 848 (22%)	9434 (21%)	5414 (22%)
D	8610 (8%)	4800 (8%)	3810 (8%)	4373 (6%)	2972 (7%)	1401 (6%)
Unknown	41 921 (38%)	22 931 (39%)	18 990 (38%)	28 454 (42%)	18 254 (42%)	10 200 (42%)
						
*Grade*						
Well differentiated	5879 (5%)	3327 (6%)	2552 (5%)	3556 (5%)	2310 (5%)	1246 (5%)
Moderately differentiated	43 371 (40%)	24 240 (41%)	19 131 (38%)	30 449 (45%)	19 981 (46%)	10 468 (43%)
Poorly differentiated	8049 (7%)	4011 (7%)	4038 (8%)	4446 (7%)	2887 (7%)	1559 (6%)
Undifferentiated	144 <1%	71 <1%	73 <1%	70 <1%	43 <1%	27 (<1%)
Unknown	51 753 (47%)	27 548 (47%)	24 205 (48%)	29 662 (44%)	18 684 (43%)	10 978 (45%)

**Table 2:** Dukes stage completeness for bowel cancer by cancer registry in England 1996–2004, and population size in 2004 for male and female patients combined aged 50–79 years

**Cancer registry**											
										**National**	
**Year of diagnosis**	**East Anglian**	**Merseyside and Cheshire**	**North West**	**Northern and Yorkshire**	**Oxford**	**South West**	**Thames**	**Trent**	**West Midlands**	**Total (%)**	**Total count**
1996	93	3	0	42	79	31	57	10	70	43	19 404
1997	94	39	0	64	84	44	56	12	70	51	19 356
1998	92	52	0	88	83	58	60	18	74	59	20 309
1999	93	35	0	88	85	58	62	52	75	62	20 217
2000	93	65	0	92	85	61	63	59	76	65	20 382
2001	93	63	0	93	82	71	62	80	76	69	19 455
2002	94	59	0	91	85	71	60	74	74	68	19 292
2003	95	69	0	89	82	76	59	57	72	66	19 328
2004	94	62	0	87	81	81	57	8	75	61	19 636
Total %	93	49	0	82	83	61	60	41	74	60	
*Total count*	*10 465*	*9428*	*15 602*	*26 287*	*8780*	*27 882*	*41 644*	*17 395*	*19 896*		*1*77 379
Population size^a^	1 675 755	1 937 893^b^		2 010 468	762 758	2 174 144	2 972 706	1 482 587	1 591 663		14 607 974

a Using boundaries for the eight cancer registries as they exist in 2009.

b Registries Merseyside and Cheshire, and North West were combined to form the North West Cancer Intelligence Service (NWCIS).
